# Genome Sequence of *Streptomyces caatingaensis* CMAA 1322, a New Abiotic Stress-Tolerant Actinomycete Isolated from Dried Lake Bed Sediment in the Brazilian Caatinga Biome

**DOI:** 10.1128/genomeA.01020-15

**Published:** 2015-09-10

**Authors:** Suikinai Nobre Santos, Ranko Gacesa, Rodrigo Gouvêa Taketani, Paul F. Long, Itamar Soares Melo

**Affiliations:** ^a^ Laboratory of Environmental Microbiology, Brazilian Agricultural Research Corporation, EMBRAPA Environment, Jaguariúna, Brazil; ^b^ Institute of Pharmaceutical Science, King’s College London, London, United Kingdom; ^c^ Faculdade de Ciências Farmacêuticas, Universidade de São Paulo, São Paulo, Brazil

## Abstract

The genome sequence of the first *Streptomyces* species isolated from the Brazilian Caatinga is reported here. Genes related to environmental stress tolerance were prevalent and included many secondary metabolic gene clusters.

## GENOME ANNOUNCEMENT

The Caatinga is one of the largest dry forest regions in the world, with many endemic species of microorganisms, plants, and animals uniquely adapted for life in this semiarid ecoregion (1). A new actinomycete designated *Streptomyces caatingaensis* CMAA 1322 was isolated from sediment collected at a dry lake bed close to the town of Remanso in the northeastern state of Bahia. Paired-end sequencing of a genomic library using an 8 single-molecule real-time (SMRT) PacBio RS SMRT cell (Pacific Biosciences BioProject no. PRJNA288757) yielded 116,269 reads, with an average length of 7,446 bp. The reads were assembled into 18 contigs, with 142.11× coverage and an *N_50_* value of 20,548 bp.

The contigs were characterized and then annotated using Rapid Annotations using Subsystems Technology (RAST) (2). The genome size was estimated to be 7,055,077 bp, containing a predicted 6,167 open reading frames (ORFs) and 413 subsystems. The G+C content was 72 mol%. The genome contained a remarkably large number of genes (135) predicted to encode proteins with functions related to stress responses, including osmotic stress, heat shock, and oxidative stress. The Caatinga ecoregion is characterized by intense heat and prolonged periods of drought or excessive rainfall and flooding. Understanding how the genome of *S. caatingaensis*, which is rich in stress response genes, enables a marked functional response to extreme ecological conditions is worthy of future research. Additional annotation using antiSMASH version 3.0 (3) identified 41 potential secondary metabolism-encoding gene clusters, which is the second largest number of gene clusters for the biosynthesis of natural products so far described to be encoded in the genome of a streptomycete (4) Products from these gene clusters included 13 nonribosomal peptide synthetases and 14 polyketide synthases. Initial studies indicate that *S. caatingaensis* and other microorganisms isolated from the extreme environments of the Caatinga can exhibit a wide variety of biological activities (5), suggesting that the Caatinga is an untapped resource for future natural product discovery.

### Nucleotide sequence accession numbers. 

This whole-genome shotgun project has been deposited at DDBJ/EMBL/GenBank under the accession no. LFXA00000000. The version described in this paper is version LFXA01000000.
